# Supplementary material for: Assigning confidence scores to homoeologs using fuzzy logic
Source: PeerJ. 2019 Jan 11;6:e6231. doi: 10.7717/peerj.6231 (PMC6330999; doi:10.7717/peerj.6231)
Supplement: Supplemental Information 1 — The table is divided into three parts: first 10 rows are homoeolog pairs randomly chosen from those inferred to have a Confidence Score below 60. The second part are 10 random pairs with a Confidence Score between 60-90. The last rows have a Confidence Score between 90–100. The first two columns contain the identifiers of the two genes in a homoeologous pair (both OMA IDs and source IDs). The remaining columns are: the chromosomes for each gene in the pair, the Synteny Score of the pair (see Materials and Methods), the Evolutionary Distance of the pair, the total number of orthologs for the pair (inferred by OMA), the average protein length of the pair, and domain hits of the sequence (from NCBI’s Conserved Domain Database). [file peerj-07-6231-s001.pdf]

ID1	ID2	Conf. Score	chr1	chr2	Synteny Score	Dist.	Total nr. homoeologs	Total nr. orthologs	Mean protein length (aa)	domains hits/possible functions
GOSHI02601/ Gh_A02G0676	GOSHI48756/ Gh_D09G1978	48.8	A02	D09	0	66	28	0	178	RVT-3 (Reverse transcriptase-like)
GOSHI21628/ Gh_A11G1093	GOSHI44856/ Gh_D08G0648	41.3	A11	D08	0	90	28	1	195	RVT-3 (Reverse transcriptase-like)
GOSHI09374/ Gh_A05G2713	GOSHI53862/ Gh_D11G2315	49.7	A05	D11	0	116	10	27	262	RVT-3 (Reverse transcriptase-like)
GOSHI15019/ Gh_A08G0950	GOSHI33021/ Gh_D03G0566	54.0	A08	D03	0	43.06	40	0	134.5	-
GOSHI22096/ Gh_A11G1573	GOSHI55207/ Gh_D12G0352	56.2	A11	D12	0.05	101	7	14	143.5	Protein of unknown function
GOSHI14826/ Gh_A08G0752	GOSHI33369/ Gh_D03G0929	54.8	A08	D03	0	41.21	40	0	134.5	Pectinacetylesterase
GOSHI04614/ Gh_A03G1032	GOSHI53774/ Gh_D11G2226	50.0	A03	D11	0	58	28	1	190.5	RVT-3 (Reverse transcriptase-like)
GOSHI14826/ Gh_A08G0752	GOSHI58528/ Gh_D13G1089	50.4	A08	D13	0	57	40	0	134.5	Pectinacetylesterase
GOSHI23509/ Gh_A12G0075	GOSHI54547/ Gh_D11G3017	56.0	A12	D11	0	107	2	16	166.5	C2 domain present in Soybean genes Regulated by Cold 2 (SRC2)-like proteins
GOSHI10085/ Gh_A05G3454	GOSHI33825/ Gh_D03G1396	48.3	A05	D03	0	69	28	2	176	RVT-3 (Reverse transcriptase-like)
**0-60 confidence random sample means:**					**.005**	**74.8**	**25.1**	**6**	**171.5**	
GOSHI20700/ Gh_A11G0138	GOSHI48086/ Gh_D09G1296	76.5	A11	D09	0.05	0.05	9	482	135.5	Ubiquitin; Ribosomal L40e family
GOSHI62374/ Gh_A02G1747	GOSHI33247/ Gh_D03G0805	86.7	scaffold 266_A02	D03	0.3	10.78	3	0	285	Basic leucine zipper (bZIP) domain of bZIP transcription factors
GOSHI22361/ Gh_A11G1851	GOSHI53708/ Gh_D11G2160	88.9	A11	D11	0.3	1.59	2	46	128	Epidermal patterning factor proteins;
GOSHI10599/ Gh_A06G0372	GOSHI39979/ Gh_D06G0325	72.1	A06	D06	0.05	18.68	8	2	312.5	N-acetylneuraminic acid mutarotase [Cell wall/membrane/envelope biogenesis];
GOSHI18425/ Gh_A09G2187	GOSHI46647/ Gh_D08G2476	77.7	A09	D08	0.1	8.28	2	70	175	Det1 complexing ubiquitin ligase;
GOSHI03115/ Gh_A02G1203	GOSHI33093/ Gh_D03G0641	75.4	A02	D03	0.05	5.58	3	75	280.5	Horseradish peroxidase and related secretory plant peroxidases
GOSHI16668/ Gh_A09G0364	GOSHI47191/ Gh_D09G0385	89.1	A09	D09	0.3	1.03	2	24	560	GRAS domain family;
GOSHI05038/ Gh_A03G1466	GOSHI32019/ Gh_D02G1931	89.4	A03	D02	0.35	3.75	7	168	259.5	Pleckstrin homology-like domain

GOSHI60941/ Gh_A06G2050	GOSHI41087 / Gh_D06G1447	74.3	scaffold 1356_A 06	D06	0	3.08	2	85	432	leucine-rich repeat receptor-like protein kinase; Provisional
GOSHI02830/ Gh_A02G0909	GOSHI48365/ Gh_D09G1581	74.4	A02	D09	0	2.37	9	1256	263	40S ribosomal protein S4; Provisional
**60-90 confidence random sample means:**					**0.15**	**5.5**	**4.7**	**220.8**	**283.1**	
GOSHI25440/ Gh_A12G2060	GOSHI57063/ Gh_D12G2238	99.8	A12	D12	0.9	1.05	2	54	671	GRAS domain family;
GOSHI13604/ Gh_A07G1635	GOSHI43705/ Gh_D07G1835	86.5	A07	D07	0.5	10.55	2	58	907.5	Catalytic domain of the Serine/Threonine kinases, Interleukin-1 Receptor Associated Kinases and related STKs;
GOSHI13021/ Gh_A07G1034	GOSHI42996 /Gh_D07G1113	92.1	A07	D07	0.6	3.15	2	64	406	Modified RING finger domain;
GOSHI17966 /Gh_A09G1716	GOSHI48599/ Gh_D09G1820	95.9	A09	D09	0.7	3.22	2	124	341	Domain of unknown function (DUF4094)
GOSHI09048/ Gh_A05G2373	GOSHI38581/ Gh_D05G2637	99.2	A05	D05	0.8	1.22	2	294	409	WD40 domain
GOSHI14012/ Gh_A07G2058	GOSHI44139/ Gh_D07G2273	92.5	A07	D07	0.65	5.83	2	22	228	Chalcone isomerase-like
GOSHI23833/ Gh_A12G0407	GOSHI55260/ Gh_D12G0406	90.3	A12	D12	0.45	5.46	4	10	289.5	Domain of unknown function (DUF313)
GOSHI13142 /Gh_A07G1158	GOSHI43137 /Gh_D07G1257	95.9	A07	D07	0.70	4.1	2	638	273	haloacid dehalogenase (HAD) superfamily
GOSHI18285/ Gh_A09G2041	GOSHI49015/ Gh_D09G2247	96.0	A09	D09	0.7	1.94	2	160	882	nuc_hydro: Nucleoside hydrolases.
GOSHI00802/ Gh_A01G0819	GOSHI28715/ Gh_D01G0847	99.8	A01	D01	0.9	1.94	2	14	367	No apical meristem (NAM) protein
**90-100 confidence random sample means:**					**0.69**	**3.85**	**2.2**	**143.8**	**477.4**	

Supplemental Table 1. Manual evaluation of a sample of homoeologous pairs in Gossypium hirsutum. The table is divided into three parts: first 10 rows are homoeolog pairs randomly chosen from those inferred to have a Confidence Score below 60. The second part are 10 random pairs with a Confidence Score between 60-90. The last rows have a Confidence Score between 90-100. The first two columns contain the identifiers of the two genes in a homoeologous pair (both OMA IDs and source IDs). The remaining columns are: the chromosomes for each gene in the pair, the Synteny Score of the pair (see Materials and Methods), the Evolutionary Distance of the pair, the total number of orthologs for the pair (inferred by OMA), the average protein length of the pair, and domain hits of the sequence (from NCBI’s Conserved Domain Database).
