# Supplementary material for: Assigning confidence scores to homoeologs using fuzzy logic
Source: PeerJ. 2019 Jan 11;6:e6231. doi: 10.7717/peerj.6231 (PMC6330999; doi:10.7717/peerj.6231)
Supplement: Supplemental Information 2 [file peerj-07-6231-s002.pdf]

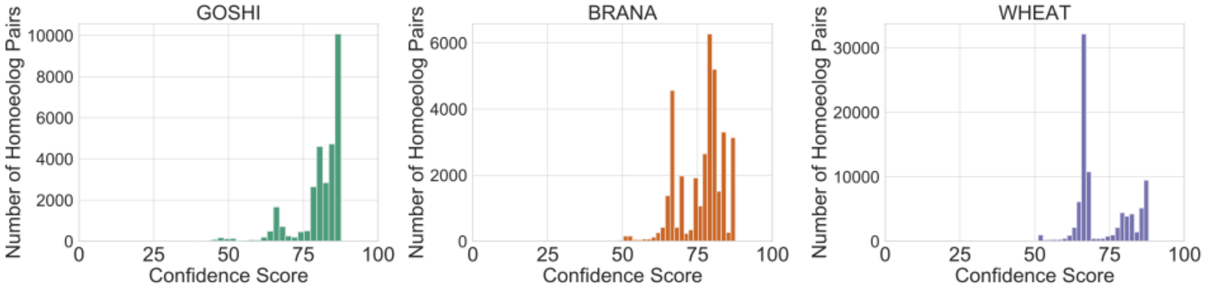

Supplementary Figure 1. Distribution of crisp confidence scores for each genome before post-processing scaling to 100 as the max confidence score.
